# Efficient Plasmid-Based Rescue of T7 RNA Polymerase-Driven Calicivirus Reverse Genetics Systems in Mammalian Cells Using Vaccinia Virus RNA-Capping Enzymes

**DOI:** 10.3390/v18050536

**Published:** 2026-05-04

**Authors:** Frazer J. T. Buchanan, Markella Loi, Charlotte Chim, ShuXian Zhou, Rebekah Penrice-Randal, Leandro X. Neves, Maximilian Erdmann, Edward Emmott

**Affiliations:** Centre for Proteome Research, Department of Biochemistry, Cell & Systems Biology, Institute of Systems, Molecular & Integrative Biology, Biosciences Building, Crown Street, University of Liverpool, Liverpool L69 7ZB, UK; f.j.t.buchanan@liverpool.ac.uk (F.J.T.B.); charlottechim4@gmail.com (C.C.); rebee@liverpool.ac.uk (R.P.-R.); l.xavier-neves@liverpool.ac.uk (L.X.N.);

**Keywords:** calicivirus, norovirus, reverse genetics, RNA capping

## Abstract

The caliciviruses include important human and animal pathogens such as norovirus, sapovirus and feline calicivirus. Viral reverse genetics is performed to understand the fundamental biology of these viruses, as well as a potential route to generate live-attenuated vaccines. Calicivirus reverse genetics systems have typically relied on either the production of in vitro-transcribed RNA or plasmid-based rescue, either from a mammalian promoter or through supplementing with helper enzymes through means of a helper virus. Here, we present a novel system integrating vaccinia capping enzymes D1R and D12L encoded on plasmids as part of a system for murine norovirus (MNV) reverse genetics. The addition of D1R, D12L and T7 RNA polymerase-expressing plasmids increases the viral titres of rescued MNV in both BSR-T7 cells and transgenic BSR-T7 cells expressing murine CD300LF (BSR-T7^CD300LF^), and viral protein abundance. When the murine norovirus receptor is expressed in BSR-T7^CD300LF^, viral titres increased 100–1000-fold compared to standard BSR-T7 cells. This system offers a robust, increased throughput means of assessing viral mutants over parallel in vitro transcription and capping reactions for multiple mutants, without requiring a helper virus.

## 1. Introduction

Human norovirus is far more than a transient “stomach bug”, manifesting as the leading cause of acute gastroenteritis worldwide, thus representing a persistent challenge to healthcare systems and a major concern to public health [1]. Transmission occurs mainly through the faecal–oral route and is facilitated by the virus’s high infectivity and rapid transmission rates [1]. These characteristics enable norovirus to spread across communities and settings such as care homes and schools, where outbreaks can place a significant strain on health care resources.

Recent epidemiological surveillance data in the United Kingdom suggest shifting patterns in norovirus activity, with notable increases in confirmed cases since 2023 and continued circulation of the virus in the following years, particularly during the winter months [2]. Such trends emphasise norovirus’s ever-changing dynamics of transmission and serve as a reminder of the significance of sustained monitoring and research efforts. With no licenced vaccines and efficient treatments, stricter hygiene practices serve as the main preventative measures against norovirus [3]. Additionally, the continuous emergence of new variants like GII.17 further complicates and delays the development of effective therapeutics [4]. Progress towards these goals relies not only on epidemiological surveillance, but also on a detailed understanding of the molecular mechanisms that modulate norovirus replication and the conserved biological events within its viral family, the *Caliciviridae*, which includes feline calicivirus (FCV) and murine norovirus (MNV).

**Figure 1:**
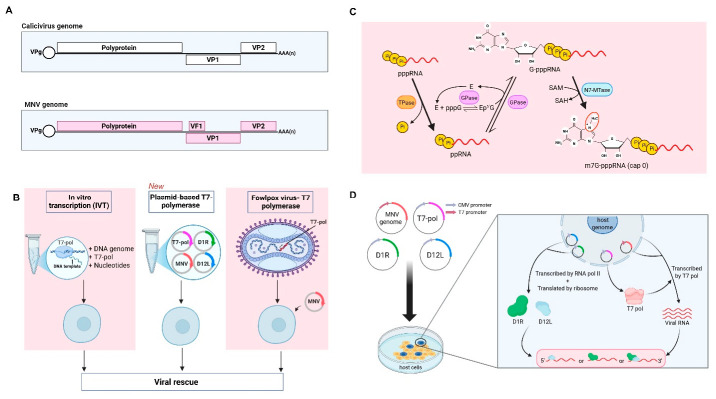
Summary of T7-based systems for Calicivirus reverse genetics. (**A**) Schematic outlining a generic calicivirus MNV genome, including polyprotein, virulence factor 1 (VF1), VP1, VP2, and (VPg). (**B**) Outline of current T7-based reverse genetics systems (pink panels): IVT and Fowlpox-T7 (FPV-T7) based rescue. The middle panel outlines a reverse genetics system incorporating vaccinia virus-capping proteins D1R and D12L. (**C**) Canonical RNA 5′ capping: triphosphorylated RNA (pppRNA) is converted to dephosphorylated RNA (ppRNA) by an RNA 5′ triphosphatase (TPase). Guanylytransferase (GTase) forms a covalent enzyme intermediate (E-pG) and transfers GMP to the 5′ end of ppRNA, generating GpppRNA. Cap structure is then methylated at the N7 position of the guanine by methyltransferase (N7-MTase) using S-adenosylmethionine (SAM) as the methyl donor, producing m^7^GpppRNA. (**D**) A combination of genome and helper plasmids (D1R, D12L and T7-pol) would allow non-canonical capping of T7 RNA transcripts produced intracellularly. The figure was generated in BioRender.

Caliciviruses are characterised by linear single-stranded, positive-sense RNA genomes of 7.4–8.3 kb, and depending on their genus, possess either two or three opening reading frames (ORFs) [5,6]. The non-structural (NS) proteins 1–7 (NS1-7) are encoded as a large polyprotein from ORF1 (~5.1 kb), beginning at the 5′ end of the genome. Genes encoding the structural proteins are located at the 3′ end of the genome, and encode major structural protein 1 (VP1) and minor capsid protein (VP2). Processing of the polyprotein is mediated by both the viral 3CL protease NS6 and host caspases; this process is essential for allowing the completion of the replication cycle [7,8] (Figure 1A). Viral protein genome-linked (VPg), once liberated from the polyprotein, serve as a primer for the norovirus genomic and subgenomic RNA, as well as a cap substitute for translation of viral proteins [9,10,11]. Although a majority of caliciviruses, including noroviruses and sapoviruses, employ exclusively VPg-dependent translation, a subset of avian caliciviruses appears to have acquired picornavirus-like internal ribosomal entry sites (IRESs) via gene transfer [12].

Despite their long-standing clinical and epidemiological significance, progress in understanding the molecular biology of caliciviruses has been comparatively slow [5]. A major limitation in calicivirus research has been the difficulty of propagating these viruses in cell culture and the lack of animal models [13,14]. Therefore, much of what is known about calicivirus biology has been derived from a small number of experimentally tractable model viruses [15]. Chief among these are FCV and MNV [16]. Especially the latter has emerged as the most widely used surrogate for human norovirus, a case attributed to MNV’s efficient replication in tissue culture and the availability of a well-established small animal model [14].

Within these model systems, the scope of experimental analysis is shaped by the available molecular tools. For positive-sense RNA viruses, and particularly caliciviruses, these systems have traditionally relied on the delivery of in vitro-transcribed (IVT) genomic RNA [17]. Full-length viral RNA is synthesised in vitro, typically using bacteriophage RNA polymerases, and transfected directly into cells that can initiate infection [18]. IVT-based reverse genetics has been instrumental in establishing foundational knowledge and remains a reliable method for generating infectious virus [19,20]. They are often considered the gold standard system for viral rescue; however, they require additional steps, such as synthesis, compared to solely plasmid-based systems. The requirement for repeated synthesis of high-quality RNA, coupled with its susceptibility to degradation and the need for careful quality control, can limit experimental throughput [18,19]. These constraints become particularly restrictive when large numbers of viral variants must be generated, making this method poorly suited to scalable or iterative experimental designs.

Reverse genetics systems have also been pivotal to advancing virological research, allowing viral rescue from purified DNA or RNA, and thus enabling the artificial engineering of viral genes [21,22]. In calicivirus research, this approach was first successfully demonstrated for FCV in 1995, confirming recovery of the virus from a genetically engineered mutant [15,23,24]. An alternative approach for calicivirus propagation, specifically MNV, uses recombinant fowlpox virus expressing T7 RNA polymerase (FPV-T7), which permits the recovery of the virus from T7-driven expression plasmids [25]. It has been established that MNV rescue poses distinct challenges compared with other caliciviruses and that vaccinia virus-based systems were incompatible with efficient MNV replication, whereas FPV-T7 provided a permissive context [25]. Although this system represents a major advance, its reliance on helper virus infection and propagation of the helper virus in primary chicken embryo fibroblasts introduces technical limitations that restrict accessibility as well as scalability. These considerations highlight the broader need for reverse genetics systems that retain biological fidelity while addressing complexity.

Plasmid-based rescue strategies could offer an alternative for initiating viral replication by allowing intracellular transcription of viral genomes from DNA templates. Here, we introduce a plasmid-based rescue system for MNV that combines a T7-driven viral genome with helper plasmids encoding the vaccinia virus mRNA capping enzyme subunit D1R and its cofactor D12L [26,27] (Figure 1). This system is designed to enhance the production of translation-competent viral RNA while avoiding the need for IVT or helper virus propagation. All components are delivered as plasmid DNA, allowing rapid exchange of viral genome constructs and facilitating experimental workflows that require repeated or parallel manipulation. This feature is particularly relevant for studies that require comparison of multiple variants and may encourage the adaptation of reverse genetics strategies to other caliciviruses that remain difficult to manipulate.

Beyond its immediate application to MNV, this strategy could provide the framework for a more accessible and scalable platform for routine viral rescue, complemented by proteomic profiling and may inform the design of other rescue systems for FCV and potentially other RNA viruses.

## 2. Materials and Methods

### 2.1. Cell Culture

Cells were tested at monthly intervals for mycoplasma and confirmed negative for mycoplasma infection. BV-2 cells were a gift from Prof. Ian Goodfellow, University of Cambridge. A BHK-21 cell line overexpressing T7 RNA polymerase (herein referred to as BSR-T7), Professor Julian Hiscox, University of Liverpool [28]. Cells were cultured in DMEM (Sigma-Aldrich, Burlington, MA, USA) supplemented with 10% Foetal Bovine Serum (FBS) (Sigma-Aldrich, Burlington, MA, USA) and 50 U mL^−1^ penicillin and 50 µg mL^−1^ streptomycin. All cells were maintained at 37 °C with 5% CO_2_. BV-2 cells were used in this study for MNV TCID_50_s. BSR-T7 cells were used in this study for viral rescue. BSR-T7 cells overexpressing the MNV receptor, murine CMRF35-like molecule 1 (CD300LF, BSR-T7^CD300LF^), positive cells, were maintained in 2.5 µg mL^−1^ of puromycin (Gibco, Waltham, MA, USA).

### 2.2. Viruses

Full-length recombinant MNV (clone based on the NC_008311.1 Norovirus GV) was generated from pT7-MNV-3′RZ, which contains the full genome under the control of a T7 polymerase promoter [25]. All MNV infections were conducted in BV-2 cells.

### 2.3. Plasmids

The vaccinia capping plasmids, pCAG-D1R and pCAG-D12L, were gifts from Takeshi Kobayashi (Addgene plasmids #89160 and #8916) [29]. A codon-optimised phage T7 RNA polymerase (T7opt) in pCAGGS plasmid was a gift from Benhur Lee (Addgene plasmid #65974) [30]. The MNV reverse genetics system pT7-MNV-3′RZ was a gift from Ian Goodfellow, University of Cambridge [25]. The pGenLenti-murine-CD300LF plasmid for lentiviral-based expression of murine CD300LF was synthesised by Genscript and is available from Addgene (Addgene plasmid #235668).

### 2.4. Generation of CD300LF-Expressing BSR-T7 Cells

BSR-T7 cells were seeded (3 × 10^5^ cells/well) into 6-well plates 24 h prior to transduction with Lentiviral vector (pGenLenti-CD300LF) (Genscript, Oxford, UK). Cells were treated for 30 min with 8 µg mL^−1^ of polybrene prior to transduction of lentivirus at an MOI of 1. Cells were then put under puromycin selection at a variable concentration of 2–4 µg mL^−1^, and cell death was monitored daily. CD300LF expression was maintained by culturing cells in 2.5 µg mL^−1^ puromycin.

### 2.5. Viral Rescue

MNV pT7-MNV-3′RZ plasmids were transfected into BSR-T7 and BSR-T7^CD300LF^ cells, alongside pCAG-D1R, pCAG-D12L, T7opt at a 1:1:1:1 ratio. BSR-T7 and BSR-T7^CD300LF^ cells were reverse-transfected using lipofectamine 2000 (Invitrogen, Waltham, MA, USA) and a total of 2 µg of plasmid DNA. After 6 h following transfection, the cell culture media were changed. Plates were then incubated for a total of 72 h. Culture plates containing the transfected cells then underwent a freeze–thaw cycle at –80 °C.

### 2.6. TCID_50_

Quantification of MNV particles was conducted via tissue culture infectious dose 50 assay in BV-2 cells. Virus was harvested by freezing cells and media at −80 °C, allowing lysis through a freeze/thaw cycle. Lysates were then serially diluted 10-fold in cell culture media and added to BV-2 cells in a 96-well plate and incubated for 120 h at 37 °C with 5% CO_2_. The TCID_50_ was calculated by determining the dilution factor required to show 50% CPE [31]. TCID_50_ results were visualised in RStudio (version 2025.09.2 + 418).

### 2.7. Western Blotting and Antibodies

Cells were lysed in 1× RIPA lysis buffer (100 mM Tris-HCl, 500 mM NaCl, 1% sodium Deoxycholate, 1% NP-40, 0.1% sodium dodecyl sulphate, ddH_2_O, supplemented with HALT cocktail protease inhibitor 1:200 (Thermo Fisher Scientific, Waltham, MA, USA) and lysates were centrifuged for 15 min at 15,000× *g*; clarified supernatants were retained in separate tubes. Samples for SDS-PAGE were prepared in 1× Laemelli buffer (BioRad, Hercules, CA, USA) supplemented with β-mercaptoethanol, 355 mM final. Proteins were separated on a 17.5% SDS-PAGE gel and subsequently transferred onto a PVDF membrane 0.45 µm (Merck Millipore, Burlington, MA, USA) using a BioRad Trans-Blot turbo transfer system. Membranes were blocked in 10% milk in Tris-buffered saline solution supplemented with 0.1% Tween (TBS-T, Sigma-Aldrich, Burlington, MA, USA). Membranes were then incubated at 4 °C overnight with gentle agitation with rabbit anti-VPg antibody (1:1000) in 5% milk-TBS-T (In-house custom polyclonal antibody generated by (Eurogentec, Seraing, Belgium). Membranes were washed three times in TBS-T prior to incubation with anti-rabbit HRP-conjugated secondary antibody (1:2000, Cell Signalling Technology, Danvers, MA, USA) in 5% milk-TBS-T. Membranes were washed three times in TBS-T and incubated in Pierce© ECL reagent (Thermo Fisher Scientific, Waltham, MA, USA) prior to imaging on a ChemiDoc MP System (BioRad, Hercules, CA, USA).

### 2.8. Proteomics

BSR-T7 cells were harvested and washed twice in pre-warmed Dulbecco’s phosphate-buffered saline (DPBS; Sigma Aldrich, Burlington, MA, USA), then lysed in 100 mM HEPES pH 7.4 containing 1% IGEPAL CA-630 (Sigma-Aldrich, Burlington, MA, USA), 1% sodium dodecyl sulphate (Sigma-Aldrich, Burlington, MA, USA), and 1× HALT protease inhibitor cocktail (Thermo Fisher Scientific, Waltham, MA, USA) and heated at 95 °C for 5 min, 900 rpm. Benzonase Nuclease (Sigma-Aldrich, Burlington, MA, USA) was added to 1.25 U µL^−1^, followed by 15 min incubation at 37 °C, then dithiothreitol (DTT, Sigma-Aldrich, Burlington, MA, USA) was added to 4 mM of the final concentration, and samples were heated at 60 °C for 10 min, at 900 rpm. The samples were allowed to cool to room temperature, and iodoacetamide (IAA, Sigma-Aldrich, Burlington, MA, USA) was added to a 14 mM final concentration following 30 min incubation at room temperature (RT) in the dark. The excess of IAA was then quenched with a second addition of 3 mM DTT to the samples. Protein concentration was measured using Pierce^TM^ BCA Protein Assay Kit (Thermo Fisher Scientific, Waltham, MA, USA) following the manufacturer’s microplate procedure. Of each sample, 15 µg was then transferred to a 96-well LoBind plate (Eppendorf, Hamburg, Germany) for SP3-based clean-up and digestion [32]. In brief, sample volumes were normalised to 50 µL with lysis buffer, then 5 µL of 30 µg µL^−1^ Sera-Mag^TM^ Carboxylate-Modified Magnetic Beads and SpeedBeads (Cytiva, Marlborough, MA, USA; #45152105050250) were added at a 10:1 bead-to-protein ratio. Protein was precipitated onto beads by adding absolute acetonitrile (MeCN, Sigma-Aldrich, Burlington, MA, USA) to a final concentration of 70%, followed by 15 min incubation, RT, 750 rpm. In a magnetic rack, beads were washed twice with absolute ethanol, followed by one wash of MeCN. To each well, 150 µL of 50 mM ammonium bicarbonate containing 3 ng µL^−1^ Trypsin Gold (Promega, Madison, WI, USA; 1:30 protease to protein ratio) was added, followed by a 2 min sonication in a water bath to disaggregate the beads. Tryptic digestion was carried out at 37 °C for 16 h, 1100 rpm. The digestion supernatant containing tryptic peptides was transferred to a fresh 96-well LoBind plate, and TFA was added to 0.3% final concentration to terminate the digestion.

LC-MS/MS analysis was carried out using an Evosep One (Evosep Biosystems, Odense, Denmark) coupled to a timsTOF HT mass spectrometer (Bruker, Billerica, MA, USA). Approximately 150 ng of the tryptic digests was loaded into EV2011 Evotip Pure tips (Evosep Biosystems, Odense, Denmark), as per the manufacturer’s instructions. The peptide mixtures were resolved using an EV1906 Endurance Column (Evosep Biosystems, Odense, Denmark; ReproSil-Pur C18, 1.9 µm, 15 cm × 150 µm), 40 °C, and the 30 samples per day (SPD) method. The mass spectrometer was equipped with a CaptiveSpray (Bruker, Billerica, MA, USA) ion source operating at 1600 V, and mass spectra (100 to 1700 *m*/*z* window) were acquired in PASEF [33] positive mode. TIMS settings included a 100 ms ramp, ion mobility (IM) coefficients (K_0_^−1^) from 0.6 to 1.6 cm^2^ V^−1^ s^−1^. Ten PASEF ramps MS/MS were scanned per cycle, and peptide precursors within the polygon filter applied to the *m*/*z* and IM plane were isolated (2 Th for *m*/*z* < 700 and 3 Th for *m*/*z >* 700) for activation by collision-induced dissociation (CID). Collision energy was linearly ramped as a function of IM, starting at 20 eV until 59 eV across 0.6 to 1.6 cm^2^ V^−1^ s^−1^. Dynamic exclusion was set to 0.4 min. The injection order was randomised using the sample() function in R.

Spectral data were processed using FragPipe v22 [34] using the “LFQ-MBR” workflow, but with MBR disabled. Digestion mode was set to semi-specific trypsin (P1 = K/R unless P1′ = P), and up to 1 missed cleavage was allowed. Target-decoy (reverse) was used to estimate the false-discovery rate (FDR), and results were filtered at 1% FDR at the PSM, peptide and protein levels. Database search was performed against UniProt’s Golden Hamster (UP000189706, 20,395 entries; downloaded on 12 November 2024) and Murine Norovirus 1 (UP000109015, 4 entries; downloaded on 8 June 2024) reference proteomes. Data analysis was performed in R using the ggplot2 package for box plots and coverage map diagrams. Data are available via ProteomeXchange with identifier PXD074707.

### 2.9. Data and Code Availability

All code for data analysis is available from https://github.com/emmottlab/T7Capping_2024 accessed on 3 May 2026. The mass spectrometry data have been submitted to ProteomeXchange Consortium (ProteomeCentral Data and Tools) via the PRIDE partner repository with the dataset identifier PXD074707. All manuscript figures are available through Figshare (https://doi.org/10.6084/m9.figshare.31812781).

## 3. Results

### 3.1. Efficient Cell-Based Rescue from T7-Driven Calicivirus Reverse Genetics Systems Requires Co-Expression of Capping Enzymes

Calicivirus reverse genetics systems fall into two categories: (1) those which are driven by the T7 RNA polymerase promoter and are either synthesised as RNA transcripts and capped in vitro or through use of FPV-T7 to drive T7 expression in cells, or (2) those where expression is driven by a mammalian promoter. The BSR-T7 cell line, which expresses T7, is commonly used for viral reverse genetics and viral protein expression [28,35,36]. In the case of mammalian promoter-driven expression and FPV-T7, either nuclear mammalian RNA-capping enzymes or fowlpox capping enzymes would be expressed within infected cells. However, in the absence of viral capping enzymes, T7 transcription would be expected to result in the synthesis of full-length, albeit uncapped mRNA. As RNA capping is well established as crucial for efficient translation of mRNA into protein, we hypothesised that expression of T7 polymerase alone would result in poor translation of viral proteins from a T7 promoter-driven viral reverse genetics system and, consequently, low efficiency of virus recovery.

**Figure 2:**
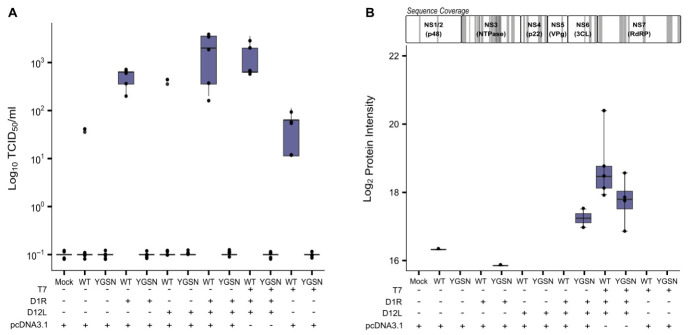
BSR-T7 cells require supplementation with T7 polymerase and both vaccinia capping enzymes for efficient MNV rescue. (**A**) BSR-T7 cells were transfected with, in total, 2 µg plasmid DNA. Combinations of plasmids are identified by +/− indicators (below plot). Plasmids were constituted as 1:1 ratio; pcDNA3.1 empty vector was used to top up amounts to 2 µg. Plates were freeze–thawed 96 h post-transfection and clarified by centrifugation. Resulting supernatant was inoculated into 96-well plate containing BV-2 cells and subsequently assayed 96 h later by TCID_50_. Y-axis represents TCID_50_ mL^−1^ log_10_. (n = 4, boxplots show median (centre line), interquartile range (box; 25th–75th percentiles), and whiskers extending to 1.5× the interquartile range). (**B**) BSR-T7 cells were transfected with, in total, 2 µg plasmid DNA. Combinations of plasmids are identified by +/− indicators (below plot). Plasmids were constituted as 1:1 ratio; pcDNA3.1 empty vector was used to top up amounts to 2 µg. Plates were freeze–thawed 72 h post-transfection and clarified. Shaded areas on diagram highlight position of peptides identified by LC-MS. Viral polyprotein abundance plotted for each condition of plasmid combination. Sequence coverage indicated origin against viral polyprotein from which peptides were derived (n = 5).

We tested the hypothesis that cell-based rescue of T7-driven caliciviruses, using MNV as a model, could be achieved by plasmid-based overexpression of T7 RNA polymerase and the vaccinia virus D1R and D12L capping enzymes as helper plasmids alongside the viral T7-driven reverse genetics plasmid. Figure 2A shows the impact of overexpression of individual T7 RNA polymerase, D1R, and D12L alongside either wild-type (WT) or an inactive polymerase mutant (YGSN) calicivirus reverse genetics plasmid encoding MNV. As expected, all conditions using the YGSN plasmid genome, a plasmid conferring a point mutation within the conserved YGDD active site of the viral RNA-dependent RNA polymerase, which prevents viral replication but can serve as a control of input translation for ORF1. As expected, the infectious virus was only recovered using the WT plasmid, while all conditions employing the YGSN plasmid failed to produce infectious virus (Figure 2A). The most efficient viral rescue required expression of all three helper plasmids (T7 + D1R + D12L), alongside the WT viral plasmid, with titres increased 2-fold log_10_ relative to the standard WT + T7 condition. Notably, the combination of WT + D1R + D12L also demonstrated an approximately two-fold log_10_ increase compared to the WT + T7 only combination. This demonstrates inclusion of the helper plasmids, including T7 RNA polymerase, increased virion production, suggesting that both supplementing the endogenous levels of T7 RNA polymerase and capping the T7-generated transcripts contribute to efficient viral rescue from the plasmid.

We next sought to evaluate the abundance of viral proteins in cells transfected with different combinations of reverse genetics and helper plasmids by LC-MS/MS (Figure 2B). Similar to Figure 2A, BSR-T7 cells were transfected with a combination of helper plasmids and MNV genome plasmid (WT or YGSN). Cells were harvested 96 h post-transfection and prepared through SP3 digest in preparation for analysis by LC-MS/MS. As expected, conditions lacking either WT or YGSN genome plasmids showed no viral polyprotein expression over the limit of detection. Viral polyprotein was detected in all transfection conditions, including the WT and YGSN genomes. The YGSN combination, including (D1R + D12L) capping enzymes, resulted in an approximately 18 log_2_ intensity score for polyprotein abundance. Similarly, the YGSN combination, including all three helper plasmids (T7 + D1R + D12L), resulted in an approximately 18.5 log_2_ intensity score for polyprotein abundance. This can be explained by the production of translation-competent transcripts in the rescue, which permit protein translation but fail to complete the full replication cycle due to the YGSN defective polymerase mutation. The WT genome rescue condition, including all three helper plasmids (T7 + D1R + D12L), resulted in an approximately 1.12 log_2_ fold increase in polyprotein abundance compared to the WT genome only. While good sequence coverage of the MNV polyprotein was achieved by LC-MS/MS analysis, viral proteins expressed from the sub-genomic viral mRNA (VP1, VP2, and VF-1) were not observed (Figure 2B).

### 3.2. Cell-Based Calicivirus Rescue Is Enhanced Using Permissive Cells

BSR-T7 cells are commonly used for viral reverse genetics based on both their expression of T7 RNA polymerase, as well as deletions that impair sensing of plasmid DNA and uncapped RNA [37]. BSR-T7 cells are not known to be permissive for MNV infection. However, MNV is able to infect a range of cell lines following expression of its cognate cellular receptor: murine CD300LF [38,39]. We next sought to understand if transducing BSR-T7 cells to stably express the murine norovirus receptor would yield a more efficient rescue system. Stable expression of murine CD300LF was achieved by lentiviral transduction to deliver CD300LF to the BSR-T7 cells under the control of the CMV promoter, alongside a puromycin-resistance cassette.

**Figure 3:**
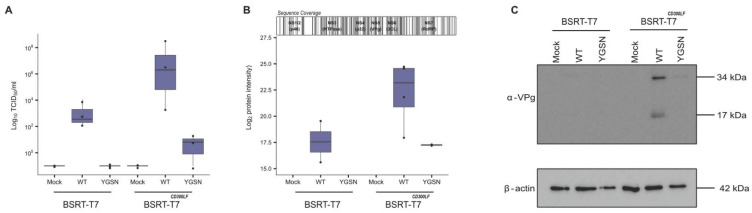
MNV rescue is enhanced by stable expression of CD300LF in BSR-T7 cells. (**A**) BSR-T7 cells were transfected with total of 2 µg plasmid DNA. This includes both D1R, D12L, T7 and either WT or YGSN MNV genome. Plates were freeze–thawed 72 h post-transfection and clarified. Resulting supernatant was inoculated into 96-well plate containing BV-2 cells and subsequently assayed 96 h later by TCID_50_. Y-axis represents log_10_ (TCID_50_ mL^−1^). (n = 3). (**B**) BSR-T7 cells were transfected with total of 2 µg plasmid DNA. Combinations of plasmids are identified by +/− indicators (below plot). Plasmids were constituted as 1:1 ratio; pcDNA3.1 empty vector was used to top up amounts to 2 µg. Plates were freeze–thawed 72 h post-transfection and clarified. Shaded areas on diagram highlight position of peptides identified by LC-MS. Viral polyprotein abundance plotted for each condition of plasmid combination. Sequence coverage indicated origin against viral polyprotein from which peptides were derived (n = 5). (**C**) BSR-T7 or BSR-T7^CD300LF^ cells transfected with either WT or YGSN MNV plasmid, as well as D12L or D1R helper plasmids, were lysed and analysed by Western blotting for viral VPg protein and a B-actin control. The 34 kDa bands in the anti-VPg plot represent precursor polyprotein containing VPg and 17 kDa fully cleaved VPg.

To test our hypothesis that expression of murine CD300LF would result in a more efficient virus rescue, we transfected the MNV genome plasmids, along with the T7 RNA polymerase, as well as D1R and D12L helper plasmids, into either wild-type BSR-T7 cells or BSR-T7^CD300LF^ cells. As can be seen in Figure 3A, expression of murine CD300LF cells greatly improves the titres obtained at 72 h post-transfection, with a 100–1000-fold increase in average titres, from mid 10^3^ TCID_50_ mL^−1^ to 10^7^ TCID_50_ mL^−1^.

In agreement with the TCID_50_ data, we observed a marked increase in VPg expression in BSR-T7 cells expressing CD300LF compared to naïve BSR-T7 cells via Western blotting; see Figure 3C. This is indicated by the stronger bands representing cleaved and uncleaved VPg (including precursors) detected with anti-VPg antibody.

Given the increase in viral protein abundance when T7 and D1R and D12L are included in the rescue setup, we set out to determine polyprotein abundance by LC-MS/MS in both BSR-T7 cells and the BSR-T7^CD300LF^ cells (Figure 3B). We observed an approximately 1.3 log_2_ fold increase in intensity for the BSR-T7^CD300LF^ cells compared to the BRST7 cells. This was expected, as the CD300LF expression permits onward replication of the virus produced in initially untransfected/infected cells. Viral protein expression from the sub-genomic viral RNA (VP1, VP2, and VF-1) was also detected (Figure S1).

## 4. Discussion

Viral rescue systems for positive-sense RNA viruses and, more specifically, caliciviruses typically rely on the delivery of the viral genome in the form of in vitro transcribed (IVT) RNA. An alternative approach involves providing a viral genome encoded within a plasmid under T7 promoter control, coupled with infection of cells using recombinant FPV-T7 [25]. Whilst this FPV-based system permits genome expression without direct RNA transfection, it requires propagation of recombinant FPV in primary chicken embryo fibroblasts, which can be challenging to access, is labour-intensive, and can introduce variability due to the primary cell context. IVT RNA-based transfections, by contrast, remain the gold standard for initiating viral replication and provide precise control over input RNA molecules. However, throughput can be limited in large-scale mutagenic screens, and efficiency can be impacted by RNA stability and host cell permissiveness. Reagents for in vitro RNA synthesis can also introduce significant expense to workflows. These limitations in current rescue systems highlight trade-offs among ease of use, scalability, expense, utility and experimental control, underscoring an avenue for alternative rescue strategies.

Plasmid-based approaches for calicivirus reverse genetics employing eukaryotic promoters have been successfully applied previously. Examples include Tet-inducible Pol II-driven murine norovirus reverse genetics [40], EF-1-α driven reverse genetics for murine norovirus [41] and feline calicivirus [42] and CMV-driven reverse genetics for RHDV [43]. These systems have the clear advantage of simplicity, using a single or pair of plasmids for transfection, and as they employ mammalian promoters, they are capped and exported from the nucleus in line with standard mRNA processing. Disadvantages include often low yields, e.g., 10^3^ [40], and the potential for cryptic splicing [44,45].

Here, we present a novel approach for plasmid-based rescue of MNV by integrating plasmids encoding the vaccinia virus-capping enzyme D1R and cofactor D12L under T7 expression. Co-transfection of these helper plasmids with a plasmid encoding the MNV genome improved viral recovery in both naïve BSR-T7 cells and those rendered permissive to MNV entry (BSR-T7^CD300LF^) (Figure 3). A YGSN mutant was incorporated to account for the initial noise of input translation from the first round of genome translation. Both our viral and proteomic assays indicated improved rescue of MNV virions when using our combination plasmid-based assay (Figure 2), as we detected both increased titres and non-structural protein expression (Figure S1). The expression of CD300LF in BSR-T7 cells itself can increase viral titres, and enables detection of viral structural proteins; however, the capping approach boosts baseline rescue in standard BSR-T7 cells (Figure 3). A benefit of the presented rescue system is the ability to rapidly screen MNV mutants; using this plasmid-based system permits the swapping of the WT plasmid in the transfection with ease. The predominant benefit of this system is that it enables the use of the same plasmid for DNA-based and IVT-based reverse genetics experiments, whilst bypassing the need for FPV-T7 helper virus. DNA-based reverse genetics enables testing of more mutants at scale, whereas IVT serves as the gold-standard approach for examining mutants that are more highly attenuated, where rescue is harder to achieve due to less efficient systems.

Given that caliciviruses utilise VPg to support replication both as a primer and cap substitute for protein synthesis, it is perhaps unsurprising that supplementing RNA capping improved viral rescue [46]. The importance of VPg or capping for successful viral replication and rescue has long been established precedent [45], highlighting the critical role of 5′ end modifications in positive-sense RNA virus biology. Beyond caliciviruses, supplementation of capping functions has also been shown to improve rescue efficiency in other positive-sense RNA viruses. For example, reovirus reverse genetics systems have been demonstrated to benefit from the presence of a fusion protein (C3P3) encoding for both T7 polymerase and the African Swine Fever Virus (ASFV) capping enzyme NP868R [47]. Interestingly, the same NP868R-T7 fusion protein has been adapted for calicivirus reverse genetics, specifically Tulane Virus (TV) [48], demonstrating the versatility of combining RNA polymerase and capping functions to improve viral rescue. These observations suggest that strategies to enhance 5′ functionality, whether through VPg or capping enzymes, can be broadly applied to improve viral rescue efficiency across multiple positive-sense RNA virus families. By expressing the vaccinia virus-capping enzymes D1R and D12L, our system supplements 5′ end capping, fulfilling a key requirement for efficient calicivirus replication and highlighting the broader utility of enhancing cap-like functions in reverse genetics strategies.

A logical next step for the development of this system would be to ensure coordinated and consistent expression of both helper plasmids and the viral genome. Currently, the need to transfect three separate helper plasmids (D1R, D12L and T7) alongside the genome plasmid presents potential issues. Namely, an increase in host cell stress, activation of DNA-sensing pathways and competition for translational machinery [49,50]. One strategy to overcome these limitations would be to consider the consolidation of all helper plasmids into a single tricistronic plasmid [51]. However, the use of four plasmid systems has precedent; our system is comparable to the multi-plasmid approach used in third-generation lentiviral production [52]. An alternative approach using IRES-based translation has been presented previously, which reduces the need for exogenous RNA capping [36]. Nevertheless, our system offers versatility with the potential to interchange the genome plasmids without requiring additional redesign of upstream sequences such as IRESs, and the ability to use the same MNV plasmid for both IVT and DNA-based reverse genetics.

The system described here has the potential to improve viral rescue efficiency of not only MNV but also other members of *Caliciviridae*, which should be investigated on a case-by-case basis. In particular, Human Sapo Virus (hSapo) has been historically difficult to propagate in cell culture due to limited receptor information and inefficient replication in standard host cell lines [53,54]. The identification of CD36 as an essential host factor for hSapo propagation [55] highlights the importance of both cellular context and host factor availability in successful virus propagation. Our system could theoretically complement CD36 overexpression, supporting improved sapovirus rescue. More broadly, combining strategies that enhance RNA stability, translation and host factor engagement may facilitate the development of enhanced reverse genetics systems for other caliciviruses.

In summary, we have presented a novel plasmid-based rescue system for MNV, which improves viral output in both BSR-T7 and BSR-T7^CD300LF^ cells in culture, and we show that modification of BSR-T7 cells to express the CD300LF receptor permits high-titre rescue from current T7-based reverse genetics plasmids. We also demonstrate the importance of viral genome capping to initiate and improve the efficiency of viral rescue. This system could be expanded into other caliciviruses, such as FCV and hSapo, and indeed to other viral families, and allows for both plasmid-based and IVT-based viral reverse genetics.

## Data Availability

All code for data analysis is available from https://github.com/emmottlab/T7Capping_2024, accessed on 3 May 2026. The mass spectrometry data have been submitted to ProteomeXchange Consortium (ProteomeCentral Data and Tools) via the PRIDE partner repository with the dataset identifier PXD074707. All manuscript figures are available through Figshare (https://doi.org/10.6084/m9.figshare.31812781).

## Supplementary Materials

The following supporting information can be downloaded at: https://www.mdpi.com/article/10.3390/v18050536/s1, Figure S1: Expression of VP1, VP2 and VF1 in BSR-T7 cells expressing CD300LF.

## References

[1] Winder N., Gohar S., Muthana M. (2022). Norovirus: An Overview of Virology and Preventative Measures. Viruses.

[2] UKHSA (2026). National Norovirus and Rotavirus Report Week 43 Report Data to Week 42 Data Up to 19 October 2025.

[3] Omatola C.A., Mshelbwala P.P., Okolo M.-L.O., Onoja A.B., Abraham J.O., Adaji D.M., Samson S.O., Okeme T.O., Aminu R.F., Akor M.E. (2024). Noroviruses: Evolutionary Dynamics, Epidemiology, Pathogenesis, and Vaccine Advances—A Comprehensive Review. Vaccines.

[4] Doh H., Lee C., Kim N.Y., Park Y.-Y., Kim E., Choi C., Eyun S. (2025). Genomic diversity and comparative phylogenomic analysis of genus Norovirus. Sci. Rep..

[5] Téllez Y.P., Ibáñez C.P., Gutiérrez-Escolano A.L., Pujol F.H., Paniz-Mondolfi A.E. (2024). Molecular Biology of Caliciviruses: Cellular and Viral Proteins Involved in the Establishment of the Infection. Emerging Viruses in Latin America: Contemporary Virology.

[6] Vinjé J., Estes M.K., Esteves P., Green K.Y., Katayama K., Knowles N.J., L’Homme Y., Martella V., Vennema H., White P.A. (2019). ICTV Virus Taxonomy Profile: Caliciviridae. J. Gen. Virol..

[7] Emmott E., De Rougemont A., Hosmillo M., Lu J., Fitzmaurice T., Haas J., Goodfellow I. (2019). Polyprotein processing and intermolecular interactions within the viral replication complex spatially and temporally control norovirus protease activity. J. Biol. Chem..

[8] Emmott E., Sweeney T.R., Goodfellow I. (2015). A Cell-based Fluorescence Resonance Energy Transfer (FRET) Sensor Reveals Inter- and Intragenogroup Variations in Norovirus Protease Activity and Polyprotein Cleavage. J. Biol. Chem..

[9] Goodfellow I., Chaudhry Y., Gioldasi I., Gerondopoulos A., Natoni A., Labrie L., Laliberté J., Roberts L. (2005). Calicivirus translation initiation requires an interaction between VPg and eIF4E. EMBO Rep..

[10] Olspert A., Hosmillo M., Chaudhry Y., Peil L., Truve E., Goodfellow I. (2016). Protein-RNA linkage and posttranslational modifications of feline calicivirus and murine norovirus VPg proteins. PeerJ.

[11] Royall E., Locker N. (2016). Translational Control during Calicivirus Infection. Viruses.

[12] Arhab Y., Pestova T.V., Hellen C.U.T. (2024). Translation of Overlapping Open Reading Frames Promoted by Type 2 IRESs in Avian Calicivirus Genomes. Viruses.

[13] Desselberger U. (2019). Caliciviridae Other Than Noroviruses. Viruses.

[14] Karst S.M. (2010). Pathogenesis of Noroviruses, Emerging RNA Viruses. Viruses.

[15] Goodfellow I., Taube S., Svensson L., Desselberger U., Greenberg H.B., Estes M.K. (2016). Chapter 3.2—Calicivirus Replication and Reverse Genetics. Viral Gastroenteritis.

[16] Peñaflor-Téllez Y., Miguel-Rodríguez C.E., Gutiérrez-Escolano A.L., Rezaei N. (2022). The Caliciviridae Family. Encyclopedia of Infection and Immunity.

[17] Hoffmann E.S., Pascali M.C.D., Neu L., Domnick C., Soldà A., Kath-Schorr S. (2024). Reverse transcription as key step in RNA in vitro evolution with unnatural base pairs††Electronic supplementary information (ESI) available. RSC Chem. Biol..

[18] Lenk R., Kleindienst W., Szabó G.T., Baiersdörfer M., Boros G., Keller J.M., Mahiny A.J., Vlatkovic I. (2024). Understanding the impact of in vitro transcription byproducts and contaminants. Front. Mol. Biosci..

[19] Lee K.H., Song J., Kim S., Han S.R., Lee S.-W. (2023). Real-time monitoring strategies for optimization of in vitro transcription and quality control of RNA. Front. Mol. Biosci..

[20] Mu X., Hur S. (2021). Immunogenicity of In Vitro -Transcribed RNA. Acc. Chem. Res..

[21] Chen H., Liu H., Peng X. (2022). Reverse genetics in virology: A double edged sword. Biosaf. Health.

[22] Zhang Y., Zhang T., Xiong Y., Zheng C., Li L., Zheng C. (2025). Reverse Genetics-Based Methodology for Molecular Virology Study. Molecular Virology: Methods and Protocols.

[23] Sosnovtsev S., Green K.Y. (1995). RNA Transcripts Derived from a Cloned Full-Length Copy of the Feline Calicivirus Genome Do Not Require VpG for Infectivity. Virology.

[24] Álvarez Á.L., Arboleya A., Abade Dos Santos F.A., García-Manso A., Nicieza I., Dalton K.P., Parra F., Martín-Alonso J.M. (2024). Highs and Lows in Calicivirus Reverse Genetics. Viruses.

[25] Chaudhry Y., Skinner M.A., Goodfellow I.G. (2007). Recovery of genetically defined murine norovirus in tissue culture by using a fowlpox virus expressing T7 RNA polymerase. J. Gen. Virol..

[26] Ramanathan A., Robb G.B., Chan S.-H. (2016). mRNA capping: Biological functions and applications. Nucleic Acids Res..

[27] Tate J., Boldt R.L., McFadden B.D., D’Costa S.M., Lewandowski N.M., Shatzer A.N., Gollnick P., Condit R.C. (2016). Biochemical analysis of the multifunctional vaccinia mRNA capping enzyme encoded by a temperature sensitive virus mutant. Virology.

[28] Buchholz U.J., Finke S., Conzelmann K.-K. (1999). Generation of Bovine Respiratory Syncytial Virus (BRSV) from cDNA: BRSV NS2 Is Not Essential for Virus Replication in Tissue Culture, and the Human RSV Leader Region Acts as a Functional BRSV Genome Promoter. J. Virol..

[29] Kanai Y., Komoto S., Kawagishi T., Nouda R., Nagasawa N., Onishi M., Matsuura Y., Taniguchi K., Kobayashi T. (2017). Entirely plasmid-based reverse genetics system for rotaviruses. Proc. Natl. Acad. Sci. USA.

[30] Yun T., Park A., Hill T.E., Pernet O., Beaty S.M., Juelich T.L., Smith J.K., Zhang L., Wang Y.E., Vigant F. (2015). Efficient Reverse Genetics Reveals Genetic Determinants of Budding and Fusogenic Differences between Nipah and Hendra Viruses and Enables Real-Time Monitoring of Viral Spread in Small Animal Models of Henipavirus Infection. J. Virol..

[31] Hwang S., Alhatlani B., Arias A., Caddy S.L., Christodoulou C., Bragazzi Cunha J., Emmott E., Gonzalez-Hernandez M., Kolawole A., Lu J. (2014). Murine Norovirus: Propagation, Quantification, and Genetic Manipulation. Curr. Protoc. Microbiol..

[32] Hughes C.S., Moggridge S., Müller T., Sorensen P.H., Morin G.B., Krijgsveld J. (2019). Single-pot, solid-phase-enhanced sample preparation for proteomics experiments. Nat. Protoc..

[33] Meier F., Brunner A.-D., Koch S., Koch H., Lubeck M., Krause M., Goedecke N., Decker J., Kosinski T., Park M.A. (2018). Online Parallel Accumulation–Serial Fragmentation (PASEF) with a Novel Trapped Ion Mobility Mass Spectrometer. Mol. Cell. Proteom..

[34] Yu F., Haynes S.E., Teo G.C., Avtonomov D.M., Polasky D.A., Nesvizhskii A.I. (2020). Fast Quantitative Analysis of timsTOF PASEF Data with MSFragger and IonQuant. Mol. Cell. Proteom..

[35] Römer-Oberdörfer A., Mundt E., Mebatsion T., Buchholz U.J., Mettenleiter T.C. (1999). Generation of recombinant lentogenic Newcastle disease virus from cDNA. J. Gen. Virol..

[36] Sandoval-Jaime C., Green K.Y., Sosnovtsev S.V. (2015). Recovery of murine norovirus and feline calicivirus from plasmids encoding EMCV IRES in stable cell lines expressing T7 polymerase. J. Virol. Methods.

[37] Habjan M., Penski N., Spiegel M., Weber F. (2008). T7 RNA polymerase-dependent and -independent systems for cDNA-based rescue of Rift Valley fever virus. J. Gen. Virol..

[38] Haga K., Fujimoto A., Takai-Todaka R., Miki M., Doan Y.H., Murakami K., Yokoyama M., Murata K., Nakanishi A., Katayama K. (2016). Functional receptor molecules CD300lf and CD300ld within the CD300 family enable murine noroviruses to infect cells. Proc. Natl. Acad. Sci. USA.

[39] Orchard R.C., Wilen C.B., Doench J.G., Baldridge M.T., McCune B.T., Lee Y.-C.J., Lee S., Pruett-Miller S.M., Nelson C.A., Fremont D.H. (2016). Discovery of a proteinaceous cellular receptor for a norovirus. Science.

[40] Ward V.K., McCormick C.J., Clarke I.N., Salim O., Wobus C.E., Thackray L.B., Virgin H.W., Lambden P.R. (2007). Recovery of infectious murine norovirus using pol II-driven expression of full-length cDNA. Proc. Natl. Acad. Sci. USA.

[41] Katayama K., Murakami K., Sharp T.M., Guix S., Oka T., Takai-Todaka R., Nakanishi A., Crawford S.E., Atmar R.L., Estes M.K. (2014). Plasmid-based human norovirus reverse genetics system produces reporter-tagged progeny virus containing infectious genomic RNA. Proc. Natl. Acad. Sci. USA.

[42] Oka T., Takagi H., Tohya Y. (2014). Development of a novel single step reverse genetics system for feline calicivirus. J. Virol. Methods.

[43] Liu G., Ni Z., Yun T., Yu B., Chen L., Zhao W., Hua J., Chen J. (2008). A DNA-launched reverse genetics system for rabbit hemorrhagic disease virus reveals that the VP2 protein is not essential for virus infectivity. J. Gen. Virol..

[44] Mäkeläinen K.J., Mäkinen K. (2007). Testing of internal translation initiation via dicistronic constructs in yeast is complicated by production of extraneous transcripts. Gene.

[45] Yunus M.A., Chung L.M.W., Chaudhry Y., Bailey D., Goodfellow I. (2010). Development of an optimized RNA-based murine norovirus reverse genetics system. J. Virol. Methods.

[46] Wei C., Farkas T., Sestak K., Jiang X. (2008). Recovery of Infectious Virus by Transfection of In Vitro-Generated RNA from Tulane Calicivirus cDNA. J. Virol..

[47] Eaton H.E., Kobayashi T., Dermody T.S., Johnston R.N., Jais P.H., Shmulevitz M. (2017). African Swine Fever Virus NP868R Capping Enzyme Promotes Reovirus Rescue during Reverse Genetics by Promoting Reovirus Protein Expression, Virion Assembly, and RNA Incorporation into Infectious Virions. J. Virol..

[48] Scribano F.J., Gebert J.T., Engevik K.A., Hayes N.M., Villanueva J., Pham S., Kaundal S., Dave J.J., Prasad B.V.V., Estes M.K. (2025). BTP2 restricts Tulane virus and human norovirus replication independent of store-operated calcium entry. J. Virol..

[49] Majerciak V., Zheng Z.-M. (2025). Induction of translation-suppressive G3BP1+ stress granules and interferon-signaling cGAS condensates by transfected plasmid DNA. hLife.

[50] Semenova N., Bosnjak M., Markelc B., Znidar K., Cemazar M., Heller L. (2019). Multiple cytosolic DNA sensors bind plasmid DNA after transfection. Nucleic Acids Res..

[51] Peeters B., De Leeuw O. (2017). A single-plasmid reverse genetics system for the rescue of non-segmented negative-strand RNA viruses from cloned full-length cDNA. J. Virol. Methods.

[52] Gill K.P., Denham M. (2020). Optimized Transgene Delivery Using Third-Generation Lentiviruses. Curr. Protoc. Mol. Biol..

[53] Oka T., Stoltzfus G.T., Zhu C., Jung K., Wang Q., Saif L.J. (2018). Attempts to grow human noroviruses, a sapovirus, and a bovine norovirus in vitro. PLoS ONE.

[54] Takagi H., Oka T., Shimoike T., Saito H., Kobayashi T., Takahashi T., Tatsumi C., Kataoka M., Wang Q., Saif L.J. (2020). Human sapovirus propagation in human cell lines supplemented with bile acids. Proc. Natl. Acad. Sci. USA.

[55] Oka T., Okemoto-Nakamura Y., Takagi H. (2025). CD36 is required for human sapovirus propagation. J. Virol..

