# Supplementary material for: Efficient Plasmid-Based Rescue of T7 RNA Polymerase-Driven Calicivirus Reverse Genetics Systems in Mammalian Cells Using Vaccinia Virus RNA-Capping Enzymes
Source: Viruses. 2026 May 4;18(5):536. doi: 10.3390/v18050536 (PMC13211761; doi:10.3390/v18050536)
Supplement: Supplementary file 1 [file viruses-18-00536-s001.zip › viruses-4241314-supplementary.pdf]

Supplementary

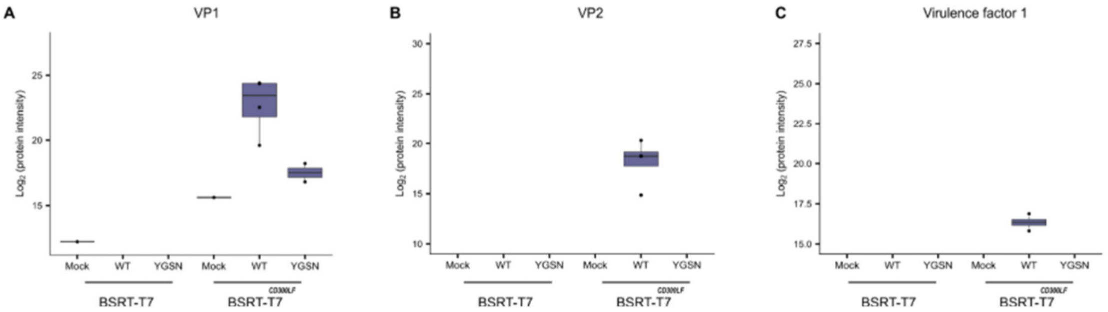

Figure S1: **Expression of VP1, VP2 and VF1 in BSR-T7 cells expressing CD300LF.**

BSR-T7 or BSR-T7^{CD300LF} cells were transfected with a total of 2 µg plasmid DNA. Including both D1R, D12L, T7 and either WT or YGSN MNV genome. Plates were freeze-thawed 72 h post-transfection and clarified. Abundance plotted for each condition of plasmid combination (n=4) for **A)** VP1, **B)** VP2 and **C)** VF1.
